# Hypoalbuminemia is associated with adverse outcomes in critically ill children with cancer

**DOI:** 10.3389/fonc.2025.1576639

**Published:** 2025-06-11

**Authors:** Vinson James, Emily Ashcraft, Cheng Cheng, Lama Elbahlawan

**Affiliations:** ^1^ Division of Pediatric Nephrology, Department of Pediatrics, LeBonheur Children’s Hospital, Memphis, TN, United States; ^2^ Department of Biostatistics, St. Jude Children’s Research Hospital, Memphis, TN, United States; ^3^ Division of Critical Care Medicine, St. Jude Children’s Research Hospital, Memphis, TN, United States

**Keywords:** intensive care unit, acute kidney injury, children with cancer, hypoalbuminemia, survival, mechanical ventilation

## Abstract

**Introduction:**

Acute kidney injury (AKI) is a serious complication encountered often in critically ill children with cancer. Hypoalbuminemia, commonly present in this population, has been associated with poor outcomes, including a higher rate of AKI. Studies examining the impact of hypoalbuminemia on outcomes in critically ill children with cancer are lacking. Therefore, the objective of this study was to investigate the impact of low serum albumin levels (SAL) on outcomes, including mortality and AKI, in critically ill children with oncologic/hematologic diseases. We also sought to examine the risk factors of AKI in this population.

**Methods:**

Retrospective review of all children with hematologic/oncologic disease admitted to the intensive care unit (ICU) from December 2020 to April 2021.

**Results:**

A total of 82 patients were included in this study cohort. The median age in our cohort was 10.3 y (0.8, 22.3), and the most common diagnosis was hematologic malignancy (41%). Thirty percent of the cohort experienced AKI; 30% of these cases were severe. Risk factors for AKI included sepsis, antiviral medications, higher nephrotoxicity index, and a higher number of nephrotoxic drugs. The rate of AKI was higher in children with SAL <2.5 g/dL (55% *vs* 27% in children with SAL ≥2.5 g/dL, *P*=0.09). SAL <3 g/dL was associated with higher rate of invasive mechanical ventilation (IMV) (15% *vs* 2% in children with SAL≥3 g/dL, *P*=0.038) and a longer duration of ICU stay (4 days *vs* 2, *P*=0.028).

**Conclusion:**

Hypoalbuminemia is associated with adverse outcomes in children with oncologic/hematologic disease. Particularly, SAL < 3 g/dL are associated with higher need for IMV and longer ICU duration. Future studies are required to investigate the impact of hypoalbuminemia in this population and whether correcting hypoalbuminemia improves outcomes.

## 1 Introduction

Acute kidney injury (AKI) is common in children with cancer, worsening outcomes in this population. Pediatric cancer patients are at heightened risk for AKI due to nephrotoxic therapies, tumor lysis syndrome, treatments such as chimeric antigen receptor T-cell (CAR-T) therapy, bone marrow transplantation, and multi-organ failure, adding significant cumulative nephrotoxic stress (1, 2). In a cohort of 1868 pediatric cancer patients, 52.6% developed AKI during treatment, highlighting the necessity of monitoring kidney function throughout the treatment process (3). Moreover, cancer therapies often require dose adjustments due to AKI, which can impair cancer treatment outcomes and overall patient prognosis. Higher risk of AKI is also observed in children admitted to the intensive care unit (ICU) with a reported prevalence of 10% to 30% (4). Previous studies have reported a correlation of hypoalbuminemia with prolonged ICU stays, higher rates of infection, and increased mortality (5). This underscores the importance of monitoring serum albumin levels as part of the clinical assessment in pediatric patients with AKI. In children with malignancies, hypoalbuminemia is common and can reflect a compromised nutritional status and systemic inflammation, which can exacerbate the course of kidney injury and lead to further complications. Studies examining the impact of hypoalbuminemia on outcomes in critically ill children with cancer are lacking. Therefore, the primary objective of our study was to investigate the impact of low serum albumin level (SAL) on outcomes, including mortality, AKI, and duration of ICU stay, in critically ill children with oncologic/hematologic diseases. The secondary objective was to examine the risk factors of AKI in this population.

## 2 Patients and methods

### 2.1 Patient population

All patients who were admitted to the ICU at St. Jude Children’s Research Hospital (SJCRH), a specialized pediatric hematologic-oncology hospital, from December 2020 to April 2021 were considered for this retrospective study. Patients were included if they met the inferred criteria of having a hematologic or oncologic diagnosis and were admitted to the ICU during the study period. Patients were excluded if they (1) were admitted to the ICU for less than 24 hours (2), were admitted solely for post-surgical recovery (3), had a pre-existing diagnosis of chronic kidney disease (CKD), or (4) lacked serum creatinine data or had substantially incomplete records.

### 2.2 Data collection

A list of all ICU admissions during the study period was obtained from institutional database queries and duplicate entries and inter-hospital transfers were removed. Comprehensive data from the EPIC electronic health record (EHR) of the patients was collected, including demographic details (age, sex, race, ethnicity, type of malignancy, history of hematopoietic cell transplant [HCT], viral infections, sepsis, graft-versus-host disease [GVHD], and thrombotic microangiopathy). Measurements of weight, length/height, and body mass index (BMI) were also recorded. In addition, the use of medications known to be nephrotoxic was recorded (**Supplementary Table 1**). The medications administered encompassed a wide range, including chemotherapeutic agents (e.g., cisplatin, carboplatin, methotrexate), antivirals (e.g., acyclovir, foscarnet), CAR-T therapy, antifungals (e.g., amphotericin B), non-steroidal anti-inflammatory drugs (NSAIDs), antibiotics (e.g., aminoglycosides, vancomycin), antihypertensives, vasopressors (e.g., epinephrine, norepinephrine), and contrast agents. In addition, Pediatric Risk of Mortality (PRISM) III and Pediatric Index of Mortality (PIM)2 data were recorded.

Laboratory data, including results of serum albumin, serum creatinine, blood urea nitrogen, cystatin C, hemoglobin, complete blood count, and liver function tests, were extracted from electronic medical records during each encounter. Laboratory values were collected on the basis of specific criteria to ensure comprehensive data analysis. The analysis included ICU preadmission laboratory results, the laboratory values associated with the highest creatinine or cystatin C levels during their ICU admission, and a value just prior to hospital discharge. If AKI occurred during the ICU admission, two additional laboratory results from the two days preceding the AKI were recorded.

AKI was diagnosed and staged according to the KDIGO criteria, which provides standardized definitions and staging for assessing the severity of kidney dysfunction (6). Stage 2 or 3 AKI was defined as severe AKI.

The nephrotoxicity index was utilized to evaluate the risk of kidney injury associated with specific drugs or drug combinations used during the study period. As part of the Nephrotoxic Injury Negated by Just-in-time Action (NINJA) framework, nephrotoxic drugs were identified and monitored to assess their impact on renal function in the study population (7, 8). NINJA’s nephrotoxic list currently contains 57 agents, whereas nephrotoxic drug score has 197 agents listed. We compared the nephrotoxic drugs administered to our patients and scored them based on these indices to evaluate patients’ cumulative risk of nephrotoxicity. The data were captured by using REDCap.

### 2.3 Statistical analysis

Descriptive statistics of patient demographics, baseline measures, and treatment outcomes are presented by whole group and by albumin cut-off values: <2.5 g/dL, <3 g/dL, <3.5 g/dL, as well as by AKI. For this analysis, the lowest SAL during the ICU admission was used. The Wilcoxon rank-sum test was used to compare differences in groups and group summaries are presented as median (min, max). The exact chi-squared test was used to examine differences in proportions by groups of interest and are presented as N (%). Simple and multiple logistic regression models were evaluated to determine the effect of albumin cut-off points on survival milestones after ICU discharge and AKI. Multivariable models controlled for serum albumin, cancer diagnosis, mechanical ventilation, BMT, vasopressor use, nephrotoxicity index, and length of ICU stay. An alpha of 0.05 was considered to be statistically significant for all statistical tests. Multiple hypothesis testing was accounted for using the Benjamini-Hochberg procedure to control the false discovery rate (FDR) at 20%, resulting in an adjusted significance threshold of alpha = 0.0381 (results presented in **Supplementary Table 2**). All analyses were performed in SAS Studio 3.83, Cary, NC, USA and FDR control was performed in RStudio 4.4.1.

## 3 Results

A total of 103 ICU admissions were initially identified. After applying exclusion criteria, 21 patients were excluded, and a final cohort of 82 patients with a diagnosed malignancy or hematologic condition were included (**Supplementary Figure 1**). The median age in our cohort was 10.3 y (0.8, 22.3), and the most common diagnosis was hematologic malignancy (41%) (**Table 1**). Sixteen patients (20%) were recipients of HCT. Nearly all patients (98%) survived beyond their ICU discharge, with 30-day, 60-day, and 1 year after discharge survival rates of 95%, 93%, and 88%, respectively. Of the 82 patients, 6% received invasive mechanical ventilation (IMV), and 13% needed vasopressor support.

**Table 1:** Risk factors for AKI.

Variable	AKI (N=25)	No AKI (N=57)	Total (N=82)	P*
Age, y	10.4 (0.8,21.6)	8.4 (0.8,22.3)	10.3 (0.8,22.3)	0.7817
Sex				
Female	11 (44)	35 (61)	46 (56)	0.1566
Male	14 (56)	22 (39)	36 (44)	
Race				
Asian	3 (12)	4 (7)	7 (9)	0.6206
African American	10 (40)	19 (33)	29 (35)	
White	12 (48)	32 (56)	44 (54)	
Other	0 (0)	2 (4)	2 (2)	
Diagnosis				
Solid tumor	4 (16)	14 (25)	18 (22)	0.3856
Leukemia/Lymphoma	13 (52)	21 (37)	34 (41)	
Brain tumor	2 (8)	11 (19)	13 (16)	
Hematologic disease	6 (24)	11 (19)	17 (21)	
HCT	6 (24)	10 (18)	16 (20)	0.5508
ICU survival	24 (96)	56 (98)	80 (98)	1
30-d survival	24 (96)	54 (95)	78 (95)	1
60-d survival	22 (88)	54 (95)	76 (93)	0.3626
1-y survival	22 (88)	50 (88)	72 (88)	1
IMV	2 (8)	3 (5)	5 (6)	1
Vasopressor	4 (16)	7 (12)	11 (13)	0.7286
Sepsis	5 (20)	3 (5)	8 (10)	0.0551
Vancomycin	10 (40)	20 (35)	30 (37)	0.8039
Antibiotics	20 (80)	46 (81)	66 (80)	1
Anti-viral	7 (28)	3 (5)	10 (12)	0.0074
Anti-fungal	11 (44)	18 (32)	29 (35)	0.3211
Diuretics	12 (48)	14 (25)	26 (32)	0.0430
Contrast	6 (24)	19 (33)	25 (30)	0.4466
Anti-hypertensive	14 (56)	20 (35)	34 (41)	0.0922
Ninja score	1.0 (0.0, 8.0)	1.0 (0.0, 6.0)	1.0 (0.0, 8.0)	0.3243
Nephrotoxicity index	3.0 (0.0, 10.0)	2.0 (0.0, 9.0)	2.0 (0.0, 10.0)	0.0842
No. nephrotoxic drugs	4.0 (0.0, 10.0)	3.0 (0.0, 8.0)	3.0 (0.0, 10.0)	0.0354
ALP (U/L)	226.0 (78.0, 543.0)	145.5 (53.0, 410.0)	163.0 (53.0, 543.0)	0.0165
ALT (U/L)	55.0 (5.0, 4487.0)	32.5 (5.0, 905.0)	36.0 (5.0, 4487.0)	0.0748
AST (U/L)	68.0 (23.0, 7000.0)	35.0 (6.0, 708.0)	45.0 (6.0, 7000.0)	0.0036
PRISM III	3.0 (0.0, 17.0)	3.0 (0.0, 20.0)	3.0 (0.0, 20.0)	0.9227
PIM2	-4.3 (-5.7, -1.6)	-4.6 (-6.4, -1.4)	-4.5 (-6.4, -1.4)	0.2775

*For group comparisons, the *P* value of nominal variables was derived from the Pearson chi-squared exact test and are presented as N (%);

The *P* value of numeric variables was derived from the Wilcoxon rank-sum test and are presented as median (min, max).

AKI, acute kidney injury; HCT, hematopoietic cell transplantation; ICU, intensive care unit; IMV, invasive mechanical ventilation; ALP, alkaline phosphatase; ALT, alanine aminotransferase; AST, aspartate aminotransferase; PRISM, Pediatric Index of Mortality; PIM, Pediatric Index of Mortality.

### 3.1 Acute kidney injury

AKI was encountered in 25 patients (30%) (**Table 1**). Diagnosis, age, BMI, weight, and HCT were not associated with any stage of AKI. However, sepsis was associated with a higher prevalence of AKI (62% in septic patients *vs*. 27% in non-septic patients, *P*=0.055). Additionally, those receiving antiviral medications were more likely to have an AKI. Other risk factors of AKI included diuretics, higher aspartate aminotransferase (AST) and alanine aminotransferase (ALT) levels, higher nephrotoxicity index, and a higher number of nephrotoxic drugs.

Of the 25 children with AKI, 8 (32%) had severe AKI, which comprised 10% of the total cohort (**Table 2**). Risk factors for severe AKI included IMV, higher AST, antiviral medications, diuretics use, higher nephrotoxicity index, and a higher number of nephrotoxic drugs. Most children (88%) with severe AKI required antihypertensive medications, whereas 36% of patients with mild/no AKI required such treatments.

**Table 2:** Risk factors for severe AKI.

Variable	AKI 0,1 (N=74)	AKI 2,3 (N=8)	Total (N=82)	P*
Age	9.3 (0.8, 22.3)	10.4 (0.8, 12.4)	10.3 (0.8, 22.3)	0.2808
Sex				
Female	42 (57)	4 (50)	46 (56)	1
Male	32 (43)	4 (50)	36 (44)	
Race				
Asian	7 (9)	0 (0)	7 (9)	0.4318
Black/African American	24 (32)	5 (63)	29 (35)	
White	41 (55)	3 (38)	44 (54)	
Other	2 (3)	0 (0)	2 (2)	
Diagnosis				
Solid tumor	18 (24)	0 (0)	18 (22)	0.0091
Leukemia/Lymphoma	31 (42)	3 (38)	34 (41)	
Brain tumor	13 (18)	0 (0)	13 (16)	
Hematologic disease	12 (16)	5 (63)	17 (21)	
HCT	13 (18)	3 (38)	16 (20)	0.3445
ICU survival	73 (99)	7 (88)	80 (98)	0.1867
30-d survival	71 (96)	7 (88)	78 (95)	
60-d survival	69 (93)	7 (88)	76 (93)	1
1-y survival	22 (88)	50 (88)	72 (88)	1
IMV	3 (4)	2 (25)	5 (6)	0.0723
Vasopressor	9 (12)	2 (25)	11 (13)	o.5896
Sepsis	7 (9)	1 (13)	8 (10)	1
Vancomycin	26 (35)	4 (50)	30 (37)	0.4555
Antibiotics	59 (80)	7 (88)	66 (80)	0.6941
Anti-viral	7 (9)	3 (38)	10 (12)	0.0536
Anti-fungal	24 (32)	5 (63)	29 (35)	0.1237
Diuretics	20 (27)	6 (75)	26 (32)	0.0110
Contrast	22 (30)	3 (38)	25 (30)	0.6946
Anti-hypertensive	27 (36)	7 (88)	34 (41)	0.0078
Ninja score	1.0 (0.0, 6.0)	2.0 (0.0, 8.0)	1.0 (0.0, 8.0)	0.2465
Nephrotoxicity index	1.8 (0.0, 9.0)	4.0 (1.5, 10.0)	2.0 (0.0, 10.0)	0.0039
No. nephrotoxic drugs	3.0 (0.0, 8.0)	6.0 (2.0, 10.0)	3.0 (0.0, 10.0)	0.0037
ALP, U/L	154.0 (53.0, 543.0)	229.0 (107.0, 395.0)	163.0 (53.0, 543.0)	0.1458
ALT, U/L	35.0 (5.0, 905.0)	104.5 (5.0, 4487.0)	36.0 (5.0, 4487.0)	0.1007
AST, U/L	36.0 (6.0, 708.0)	87.5 (33.0, 7000.0)	45.0 (6.0, 7000.0)	0.0115
PRISM III	3.0 (0.0, 20.0)	4.0 (0.0, 17.0)	3.0 (0.0, 20.0)	0.5835
PIM2	-4.6 (-6.4, -1.4)	-4.2 (-4.7, -2.5)	-4.5 (-6.4, -1.4)	0.1622

*For group comparisons, the *P* value of nominal variables was derived from the Pearson chi-squared exact test and are presented as N(%);

The *P* value of numeric variables was derived from the Wilcoxon rank-sum test and are presented as median(min, max).

AKI, acute kidney injury; HCT, hematopoietic cell transplantation; ICU, intensive care unit; IMV, invasive mechanical ventilation; ALP, alkaline phosphatase; ALT, alanine aminotransferase; AST, aspartate aminotransferase; PRISM, Pediatric Index of Mortality; PIM, Pediatric Index of Mortality.

### 3.2 Hypoalbuminemia and outcome

Out of the 82 patients, 3 lacked SAL data and were therefore excluded from this analysis. Three categories of SAL (all in g/dL) were analyzed: <2.5 *vs*. ≥2.5, <3 *vs*. ≥3, and <3.5 *vs*. ≥3.5. Fourteen percent had SAL <2.5 g/dL, whereas 33% and 65% had SAL <3 and <3.5 g/dL, respectively. Survival rate did not differ with SAL level at the time of ICU discharge. However, in children with SAL<2.5 g/dL, there was a non-statistically significant trend of lower survival rate at 30 days after ICU discharge (82% *vs* 97% in children with SAL ≥2.5 g/dL, *P*=0.09). Additionally, children with SAL < 3 g/dL had a non-statistically significant trend of lower 60-day survival rates (85% *vs* 96% in children with SAL ≥3 g/dL, *P*=0.088).

Severe AKI was diagnosed in 27% of patients with SAL <2.5 g/dL and 7% of those with SAL ≥2.5 g/dL (*P*=0.077) (**Table 3**). In addition, a non-statistically significant trend of higher any stage AKI was observed in children with SAL <2.5 g/dL (55% *vs*. 27% in children with SAL ≥2.5 g/dL, *P*=0.09). For patients with SAL <3 g/dL, severe AKI was diagnosed in 15% of the cohort *vs* 7.5% in children with SAL ≥3 g/dL. Although the prevalence of AKI was not significantly higher in patients with SAL <3 g/dL compared to those with ≥ 3 g/dL, other ICU outcome measures were impacted with these SAL (**Table 3**). Five patients received IMV, 4 of whom (80%) had SAL <3 g/dL (*P*=0.038): these patients experienced a longer duration of ICU admission (median, 4 days *vs* 2 days, *P*=0.028).

**Table 3:** Serum albumin levels (g/dL) and outcome.

Variables	2.5	≥2.5	<3	≥3	P^1^*	P^2^*
	(N=11)	(N=68)	(N=26)	(N=53)		
Age, y	6.8 (2.3, 19.2)	11.0 (0.8, 22.3)	10.9 (1.7, 22.3)	10.1 (0.8, 22.3)	0.3876	0.7902
Sex						
Female	7 (64)	36 (53)	16 (62)	27 (51)	0.5382	0.4726
Male	4 (36)	32 (47)	10 (38)	26 (49)		
HCT	1 (9)	15 (22)	4 (15)	12 (23)	0.4457	0.5594
Weight, kg	24.9 (14.6, 92.2)	34.7 (7.4, 150.0)	36.8 (10.2, 129.5)	30.7 (7.4, 150.0)	0.6152	0.6128
AKI, mild *vs*. severe						
AKI 0,1	8 (73)	63 (93)	22 (85)	49 (92)	0.0772	0.428
AKI 2,3	3 (27)	5 (7)	4 (15)	4 (8)		
AKI, none *vs*. any						
AKI	6 (55)	19 (28)	11 (42)	14 (26)	0.0927	0.1996
No AKI	5 (45)	49 (72)	15 (58)	39 (74)		
IMV	0 (0)	5 (7)	4 (15)	1 (2)	0.6025	0.0381
Vasopressor support	1 (9)	10 (15)	4 (15)	7 (13)	0.7001	1
ICU LOS, days	2.0 (1.0, 13.0)	2.5 (1.0, 20.0)	4.0 (1.0, 20.0)	2.0 (1.0, 11.0)	0.884	0.0279
ICU survival	11 (100)	66 (97)	25 (96)	52 (98)	1	1
30-d survival	9 (82)	66 (97)	23 (88)	52 (98)	0.0911	0.1017
60-d survival	9 (82)	64 (94)	22 (85)	51 (96)	0.1937	0.0875
1-y survival	9 (82)	60 (88)	21 (81)	48 (91)	0.6237	0.2836
Survival post ICU D/C (d)	23.0 (10.0, 586.0)	259.5 (0.0, 966.0)	32.0 (0.0, 586.0)	443 (0.0, 966.0)	0.4015	0.0436
Ninja score	2.0 (0.0, 4.0)	1.0 (0.0, 8.0)	2.0 (0.0, 5.0)	1.0 (0.0, 8.0)	0.0631	0.0712
Nephrotoxicity index	2.5 (0.0, 10.0)	2.0 (0.0, 9.5)	3.0 (0.0, 10.0)	1.5 (0.0, 9.5)	0.4922	0.0355
No. nephrotoxic drugs	3.0 (2.0, 7.0)	3.0 (0.0, 10.0)	4.0 (1.0, 10.0)	3.0 (0.0, 10.0)	0.6618	0.0179
PRISM III	2.0 (0.0, 20.0)	3.0 (0.0, 18.0)	3.0 (0.0, 20.0)	3.0 (0.0, 17.0)	0.3762	0.5860
PIM2	-4.4 (-5.5, -2.7)	-4.6 (-6.4, -1.4)	-4.4 (-5.8, -1.4)	-4.6 (-6.4, -1.6)	0.3705	0.1084

*For group comparisons, the *P* value of nominal variables was derived from the Pearson chi-squared exact test and are presented as N (%); the *P* value of numeric variables was derived from the Wilcoxon rank-sum test and are presented as median (min, max).

*P* ^1^ represents statistical comparisons of <2.5 *vs*. ≥2.5.

*P* ^2^ represents statistical comparisons of <3 *vs*. ≥3.

HCT, hematopoietic cell transplant; AKI, acute kidney injury; IMV, invasive mechanical ventilation; ICU, intensive care unit; LOS, length of stay; D/C, discharge; PRISM, Pediatric Index of Mortality; PIM, Pediatric Index of Mortality.

## 4 Discussion

Acute kidney injury (AKI) is a significant and common complication in pediatric patients with malignancies and hematological conditions, particularly those requiring admission to the ICU. AKI developed in one-third of our cohort, with 10% experiencing severe AKI. The etiology of AKI in these patients is often multifactorial, arising from factors such as renal hypoperfusion, nephrotoxicity from medications, and complications related to the underlying disease. In our cohort, sepsis contributed to a higher occurrence of AKI (62%). The rate of AKI observed in septic patients in our cohort is similar to that reported by Bagshaw et al. (64.4%) in a large cohort of 4,532 critically ill adult patients with septic shock (9). Additionally, nephrotoxic drug exposure was significantly associated with AKI in our cohort. Both nephrotoxicity index and the number of nephrotoxic drugs were higher in children with any AKI as well as in those with severe AKI. Indeed, previous studies have demonstrated that the odds of developing AKI are 1.5 times more likely for patients who receive nephrotoxic medication and are increased with the use of more than one nephrotoxin (7, 10). Nephrotoxic drug exposure continues to be a challenging problem in an immune-compromised population that is at higher risk of infection. The use of medications such as antibiotics, antifungals, and antivirals is inevitable with suspected infection. It is prudent to monitor renal function closely and use early prediction systems such as electronic medical record alerts to reduce the risk of AKI, especially when patients are receiving more than one nephrotoxic drug.

Hypoalbuminemia is highly prevalent in critically ill children (up to 67%) (11). After infancy, normal serum albumin levels rise to 3.4–5.6 g/dL, and values below 3.3 g/dL at any age are considered hypoalbuminemia. In our cohort, hypoalbuminemia was common and SAL <3.5 g/dL was present in 65%. Importantly, low SAL correlates with increased morbidity and mortality across hospitalized populations (12). Albumin, a protein synthesized by the liver, is critical for maintaining oncotic pressure and vascular integrity. Hypoalbuminemia occurs in children with malignancy secondary to poor nutritional status, impaired hepatic synthesis, increased catabolism, or increased loss due to capillary leak or proteinuria. Importantly, hypoalbuminemia increases the risk of mortality in critically ill patients and an increase of 1.0 g/dL in SAL at admission can result in a 73% reduction in the risk of mortality (13). **Table 4** depicts a summary of studies that examined the effect of hypoalbuminemia on survival in critically ill patients (11, 13–22). In our cohort, children with SAL <2.5 g/dl were less likely to survive for 30 days after ICU discharge and those with SAL <3 g/dl were less likely to survive for 60 days after ICU discharge.

**Table 4:** Serum albumin level and mortality in critically ill patients.

Author, year	Population (n)	Main findings
Tiwari et al. 2014 (22)	Critically ill children (435)	Incidence of hypoalbuminemia (SAL<2.5g/dL) on admission was 21% Children with SAL<2.5 g/dL had prolonged ICU stay, higher need for MV, and higher risk of mortality (25.6% *vs*. 17.7%)
Leite et al. 2016 (13)	Critically ill children (271)	Incidence of hypoalbuminemia (SAL<3.5 g/dL) on admission was 64.2% An increase of 1.0 g/dL in SAL at admission resulted in a 73% reduction in the hazard of death
Kittisakmontri et al. 2016 (21)	Critically ill children (202)	Incidence of hypoalbuminemia at admission was 57.9%
Kim et al. 2017 (20)	Critically ill children (431)	Incidence of hypoalbuminemia was 55% Children with SAL <3.5 g/dL had higher 28-d mortality (24.6% versus 9.28%)
Kendall et al. 2019 (19)	Critically ill adults with sepsis (577)	Survival probability decreased by 63.4% when admission SAL is ≤ 2.45 g/dl, and by 76.4% when the lowest SAL is ≤ 1.45 g/dl.
Nour et al. 2021 (18)	Critically ill adults (60)	At 24 h, the absence of elevated levels of microalbuminuria is strongly predictive of ICU survival
Bekhit et al. 2021 (17)	Critically ill children (100)	Incidence of hypoalbuminemia (SAL<3.5 g/dL) was 26% Higher mortality rate in children with SAL <3.5 g/dL (42.3% *vs*. 17.6%) Each unit of increase in SAL decreased the risk of mortality by 28.9%
Zhang et al. 2022 (16)	Critically ill children (9,123)	In patients with SAL < 43.2 g/L, each increase of 1 g/L in SAL was associated with an 8.1% reduction in the risk of ICU mortality
Jin et al. 2022 (15)	Critically ill adults (18,353)	Patients with SAL <30 g/l had higher ICU and hospital mortalities than those with SAL ≥30 g/l.
Ghimire et al. 2023 (14)	Critically ill adults (78)	Non-survivors had lower serum albumin levels on d3 and d5 than survivors did
Gowa et al. 2023 (11)	Critically ill children (110)	Incidence of hypoalbuminemia (SAL <3.3 g/dL) is 67.3% at 24 h The risk of mortality was 4.1 times higher in patients with hypoalbuminemia

SAL, serum albumin level; ICU, intensive care unit; MV, mechanical ventilation.

In our cohort, patients with SAL <2.5 g/dL were observed to have a trend of increased risk of any stage AKI and of severe AKI (stage 2,3). In the context of AKI, low albumin levels can exacerbate fluid shifts and lead to further renal damage. Low SAL may not only signify poor nutritional status but also contribute to the development of AKI by impairing renal perfusion and increasing the risk of nephrotoxicity. Monitoring SAL in this patient population could provide an early indication of AKI risk. Several studies, including those by Yu et al. (2017) and Wiedermann et al. (2010), have linked hypoalbuminemia to an increased risk of AKI, particularly in patients undergoing treatment with contrast media or during intensive care (23, 24). For instance, Yu et al. found that low serum albumin levels at hospital admission were predictive of AKI development in cancer patients undergoing CT with contrast (24). A meta-analysis by Wiedermann et al. (2010) that included 3,917 patients found that lower SAL was an independent predictor both of AKI and of death after AKI development. Each 1g/dL decrease in SAL increased the odds of AKI by 134% and the odds of mortality by 147% (pooled OR, 2.47) (23). Another meta-analysis of 39 studies in adult patients (n=168,740) found that each 1.0 g/dL decrease in SAL was significantly associated with an increased risk of AKI (OR,1.685) and a higher mortality in AKI patients with hypoalbuminemia (OR, 1.183) (25).

As in other studies (13, 14, 22), in our cohort, a higher requirement for IMV and longer ICU duration were observed in children with SAL <3 g/dL. In a cohort of 435 critically ill children, patients with SAL<2.5 g/dL had longer ICU stay (13.8 *vs*. 6.7 days, *P* < 0.001) and higher likelihood of respiratory failure requiring mechanical ventilator (OR, 13.7) (22).

It is unclear whether hypoalbuminemia is causally related to poor prognosis or is just a marker of disease severity. However, it is biologically plausible that it contributes to worse outcomes in critically ill children with oncologic/hematologic diseases. In addition to its role in regulating osmotic pressure and capillary membrane permeability, albumin helps to preserve endothelial cell function from oxidative stress by scavenging free radicals (26). This is particularly important in the oncological population as endothelial injury occurs often. Additionally, capillary leaks are encountered in this population in the context of sepsis or as a complication of post-hematopoietic cell transplant therapy.

This study has several limitations that warrant careful consideration. First, selection bias may limit the generalizability of our findings. As the study was conducted at a highly specialized pediatric hematologic-oncology center with advanced resources and subspecialty care; the patient population, clinical decision-making, and access to interventions may not reflect those in general or community-based pediatric ICUs. Therefore, extrapolation of results to broader settings should be done cautiously. Second, measurement bias may have influenced our findings. Serum albumin levels were collected as part of routine clinical care, and variability in timing of measurement, assay methods could have introduced inconsistencies. Third, although an association between hypoalbuminemia and adverse outcomes was observed, we caution against inferring a causal relationship, given the observational design of our study, which limits the ability to control unmeasured confounders and establish temporality or causality. Although we recognize the study’s limitation, it is important to highlight that this is the first study to investigate the effects of hypoalbuminemia in critically ill children with oncologic or hematologic conditions. Future prospective, multicenter research is needed to confirm our findings, further explore this association in such a vulnerable population, and determine whether correcting hypoalbuminemia improves outcome.

## 5 Conclusion

To conclude, hypoalbuminemia is common in critically ill children with oncologic or hematologic conditions. Hypoalbuminemia was associated with adverse outcomes such as the need for IMV and longer ICU duration. Future studies are needed to confirm these risks and to investigate whether correction by exogenous albumin infusions improves outcomes.

## Data Availability

The original contributions presented in the study are included in the article/**Supplementary Material**. Further inquiries can be directed to the corresponding author.
