# Supplementary figures and images for: Hypoalbuminemia is associated with adverse outcomes in critically ill children with cancer
Source: Front Oncol. 2025 Jun 11;15:1576639. doi: 10.3389/fonc.2025.1576639 (PMC12187846; doi:10.3389/fonc.2025.1576639)

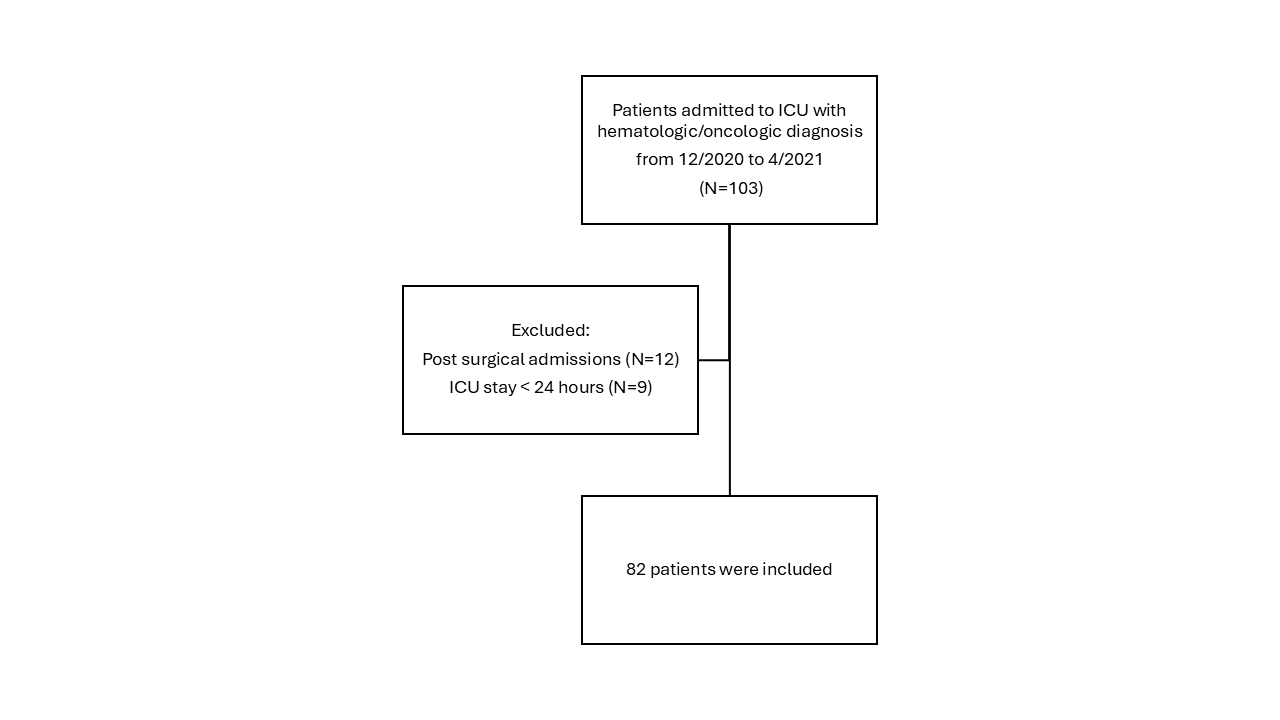

Supplement: Supplementary file 1 [file Image1.png]
