# Supplementary material for: Hypoalbuminemia is associated with adverse outcomes in critically ill children with cancer
Source: Front Oncol. 2025 Jun 11;15:1576639. doi: 10.3389/fonc.2025.1576639 (PMC12187846; doi:10.3389/fonc.2025.1576639)
Supplement: Supplementary file 2 [file Table1.docx]

Supplemental Table 1. List of nephrotoxic medications

| Acyclovir | Enalaprilat | Mesalamine |
| --- | --- | --- |
| AmBisome | Foscarnet | Methotrexate |
| Amikacin | Gadopentetate dimeglumine | Nafcillin |
| Amphotericin B | Gadoxetate disodium | Piperacillin/tazobactam |
| Captopril | Ganciclovir | Piperacillin |
| Carboplatin | Gentamicin | Sirolimus |
| Cefotaxime | Ibuprofen | Sulfasalazine |
| Ceftazidime | Ifosfamide | Tacrolimus |
| Cefuroxime | Iodixanol | Ticarcillin/clavulanic acid |
| Cidofovir | Iohexol | Tobramycin |
| Cisplatin | Iopamidol | Topiramate |
| Colistimethate | Ioversol | Valacyclovir |
| Cyclosporine | Ketorolac | Valganciclovir |
| Dapsone | Lisinopril | Vancomycin |
| Enalapril | Lithium | Zonisamide |
