# Supplementary material for: Hypoalbuminemia is associated with adverse outcomes in critically ill children with cancer
Source: Front Oncol. 2025 Jun 11;15:1576639. doi: 10.3389/fonc.2025.1576639 (PMC12187846; doi:10.3389/fonc.2025.1576639)
Supplement: Supplementary file 3 [file Table2.docx]

Supplemental Table 2. Benjamini-Hochberg False Discovery Rate Results

| **Variable** | **Comparison group** | **test** | ***P* value** | **BH FDR** |
| --- | --- | --- | --- | --- |
| Immunosuppressants | Cancer dx | chi-square | 0.0001 | 0.0103 |
| Anti-fungal | Cancer dx | chi-square | 0.0001 | 0.0103 |
| Total number of nephrotoxic drugs used | Cancer dx | WRS | 0.0007 | 0.0330 |
| Max K | Cancer dx | WRS | 0.0008 | 0.0330 |
| Max protein | Cancer dx | WRS | 0.0008 | 0.0330 |
| Maximum Creatine | AKI none vs any | WRS | 0.0011 | 0.0378 |
| Race | Cancer dx | chi-square | 0.0016 | 0.0412 |
| anitbiotics | Albumin <3 | chi-square | 0.0019 | 0.0412 |
| Survived 180 days | Cancer dx | chi-square | 0.0020 | 0.0412 |
| Survived 1 year | Cancer dx | chi-square | 0.0020 | 0.0412 |
| ICU survival | Cancer dx | chi-square | 0.0025 | 0.0468 |
| BMI | Cancer dx | WRS | 0.0028 | 0.0473 |
| Diuretics | Cancer dx | chi-square | 0.0034 | 0.0473 |
| AST | AKI none vs any | WRS | 0.0036 | 0.0473 |
| Total number of nephrotoxic drugs  used | AKI mild vs severe | WRS | 0.0037 | 0.0473 |
| Survived 60 days | Cancer dx | chi-square | 0.0037 | 0.0473 |
| Nephrotoxicity Index | AKI mild vs severe | WRS | 0.0039 | 0.0473 |
| Min  CO2 | AKI mild vs severe | WRS | 0.0053 | 0.0607 |
| Anti-virals | AKI none vs any | chi-square | 0.0074 | 0.0730 |
| Max  albumin | Cancer dx | WRS | 0.0074 | 0.0730 |
| Maximum  Creatine | AKI mild vs severe | WRS | 0.0077 | 0.0730 |
| Anti-hypertensive | AKI mild vs severe | chi-square | 0.0078 | 0.0730 |
| Cancer  diagnosis | AKI mild vs severe | chi-square | 0.0091 | 0.0798 |
| Max  K | AKI mild vs severe | WRS | 0.0093 | 0.0798 |
| Diuretics | AKI mild vs severe | chi-square | 0.0110 | 0.0877 |
| BMT | Cancer dx | chi-square | 0.0114 | 0.0877 |
| AST | AKI mild vs severe | WRS | 0.0115 | 0.0877 |
| AST | Cancer dx | WRS | 0.0150 | 0.1104 |
| Cystatin  C | AKI mild vs severe | WRS | 0.0156 | 0.1108 |
| ALP | AKI none vs any | WRS | 0.0165 | 0.1133 |
| Total number of AKI drugs received | Albumin <3 | WRS | 0.0179 | 0.1189 |
| Anti-hypertensive | Cancer dx | chi-square | 0.0194 | 0.1230 |
| Mean  albumin | Cancer dx | WRS | 0.0197 | 0.1230 |
| Total number of AKI drugs received | Albumin <3.5 | WRS | 0.0212 | 0.1284 |
| Min  CO2 | Cancer dx | WRS | 0.0242 | 0.1424 |
| PICU  time  (days) | Albumin <3 | WRS | 0.0279 | 0.1597 |
| Max  K | AKI none vs any | WRS | 0.0339 | 0.1875 |
| Total  number  of  nephrotoxic  drugs  used | AKI none vs any | WRS | 0.0354 | 0.1875 |
| Nephrotoxicity Index | Albumin <3 | WRS | 0.0355 | 0.1875 |
| Mechanical  ventilation | Albumin <3 | chi-square | 0.0381 | 0.1962 |
| Max  CO2 | Cancer dx | WRS | 0.0420 | 0.2089 |
| Diuretics | AKI none vs any | chi-square | 0.0430 | 0.2089 |
| Survival  time  after  ICU  discharge | Albumin <3 | WRS | 0.0436 | 0.2089 |
| Min  protein | Cancer dx | WRS | 0.0483 | 0.2244 |
| BMI  centile | Cancer dx | WRS | 0.0497 | 0.2244 |
| Ethnicity | AKI none vs any | chi-square | 0.0501 | 0.2244 |
| Nephrotoxicity Index | Albumin <3.5 | WRS | 0.0516 | 0.2262 |
| Anti-virals | AKI mild vs severe | chi-square | 0.0536 | 0.2270 |
| Min  phos | AKI mild vs severe | WRS | 0.0547 | 0.2270 |
| Sepsis | AKI none vs any | chi-square | 0.0551 | 0.2270 |
| Ninja  score  (excluding  Iodixanol,  Polymyxin) | Albumin <2.5 | WRS | 0.0584 | 0.2359 |
| Ninja  score  (all  drugs) | Albumin <2.5 | WRS | 0.0631 | 0.2500 |
| Ninja  score  (all  drugs) | Albumin <3 | WRS | 0.0712 | 0.2758 |
| Mechanical  ventilation | AKI mild vs severe | chi-square | 0.0723 | 0.2758 |
| ALT | AKI none vs any | WRS | 0.0748 | 0.2802 |
| AKI  mild  vs  severe | Albumin <2.5 | chi-square | 0.0772 | 0.2815 |
| anitbiotics | Albumin <3.5 | chi-square | 0.0779 | 0.2815 |
| Survived  30  days | Cancer dx | chi-square | 0.0828 | 0.2940 |
| Nephrotoxicity Index | AKI none vs any | WRS | 0.0842 | 0.2940 |
| Survived  60  days | Albumin <3 | chi-square | 0.0875 | 0.2984 |
| Survived  30  days | Albumin <2.5 | chi-square | 0.0911 | 0.2984 |
| Max  phos | AKI mild vs severe | WRS | 0.0918 | 0.2984 |
| Anti-hypertensive | AKI none vs any | chi-square | 0.0922 | 0.2984 |
| AKI  None  vs  any | Albumin <2.5 | chi-square | 0.0927 | 0.2984 |
| Baseline  Creatine | AKI mild vs severe | WRS | 0.0973 | 0.3036 |
| Ninja  score  (excluding  Iodixanol,  Polymyxin) | Albumin <3 | WRS | 0.1001 | 0.3036 |
| Max  albumin | AKI mild vs severe | WRS | 0.1003 | 0.3036 |
| ALT | AKI mild vs severe | WRS | 0.1007 | 0.3036 |
| Survived  30  days | Albumin <3 | chi-square | 0.1017 | 0.3036 |
| anitbiotics | Albumin <2.5 | chi-square | 0.1080 | 0.3178 |
| Ethnicity | Cancer dx | chi-square | 0.1141 | 0.3299 |
| Max  phos | Cancer dx | WRS | 0.1166 | 0.3299 |
| Min  CO2 | AKI none vs any | WRS | 0.1180 | 0.3299 |
| ALT | Cancer dx | WRS | 0.1185 | 0.3299 |
| Anti-fungal | AKI mild vs severe | chi-square | 0.1237 | 0.3398 |
| Sepsis | Cancer dx | chi-square | 0.1337 | 0.3624 |
| ALP | AKI mild vs severe | WRS | 0.1458 | 0.3885 |
| anitbiotics | Cancer dx | chi-square | 0.1471 | 0.3885 |
| Immunosuppressants | AKI none vs any | chi-square | 0.1520 | 0.3964 |
| Ninja  score  (excluding  Iodixanol,  Polymyxin) | Albumin <3.5 | WRS | 0.1546 | 0.3981 |
| Gender | AKI none vs any | chi-square | 0.1566 | 0.3983 |
| Ninja  score  (all  drugs) | Albumin <3.5 | WRS | 0.1590 | 0.3994 |
| Ninja  score  (excluding  Iodixanol,  Polymyxin) | AKI mild vs severe | WRS | 0.1745 | 0.4301 |
| Cystatin  C | AKI none vs any | WRS | 0.1754 | 0.4301 |
| ICU  survival | AKI mild vs severe | chi-square | 0.1867 | 0.4494 |
| Maximum  Creatine | Cancer dx | WRS | 0.1876 | 0.4494 |
| Survived  60  days | Albumin <2.5 | chi-square | 0.1937 | 0.4575 |
| Anti-virals | Cancer dx | chi-square | 0.1960 | 0.4575 |
| AKI  None  vs  any | Albumin <3 | chi-square | 0.1996 | 0.4575 |
| Nephrotoxicity Index | Cancer dx | WRS | 0.1999 | 0.4575 |
| BMI  category | Cancer dx | chi-square | 0.2268 | 0.5134 |
| ALP | Cancer dx | WRS | 0.2293 | 0.5134 |
| BMI  centile | AKI none vs any | WRS | 0.2345 | 0.5194 |
| Ninja  score  (all  drugs) | AKI mild vs severe | WRS | 0.2465 | 0.5330 |
| Survival  time  after  ICU  discharge | Albumin <3.5 | WRS | 0.2475 | 0.5330 |
| Min  phos | Cancer dx | WRS | 0.2484 | 0.5330 |
| Gender | Cancer dx | chi-square | 0.2543 | 0.5354 |
| Weight | Cancer dx | WRS | 0.2547 | 0.5354 |
| BMI | AKI mild vs severe | WRS | 0.2605 | 0.5421 |
| Admission  Creatine | AKI mild vs severe | WRS | 0.2677 | 0.5485 |
| Baseline  Creatine | Cancer dx | WRS | 0.2709 | 0.5485 |
| Min  albumin | Cancer dx | WRS | 0.2716 | 0.5485 |
| Age | AKI mild vs severe | WRS | 0.2808 | 0.5564 |
| Survived  180  days | Albumin <3 | chi-square | 0.2836 | 0.5564 |
| Survived  1  year | Albumin <3 | chi-square | 0.2836 | 0.5564 |
| Max  protein | AKI mild vs severe | WRS | 0.2981 | 0.5793 |
| Ethnicity (binary) | AKI none vs any | chi-square | 0.3165 | 0.6068 |
| Cystatin  C | Cancer dx | WRS | 0.3208 | 0.6068 |
| Anti-fungal | AKI none vs any | chi-square | 0.3211 | 0.6068 |
| Ninja  score  (all  drugs) | AKI none vs any | WRS | 0.3243 | 0.6073 |
| Ninja  score  (excluding  Iodixanol,  Polymyxin) | Cancer dx | WRS | 0.3294 | 0.6113 |
| Weight | AKI mild vs severe | WRS | 0.3326 | 0.6117 |
| Max  albumin | AKI none vs any | WRS | 0.3420 | 0.6171 |
| Survived  30  days | AKI mild vs severe | chi-square | 0.3421 | 0.6171 |
| BMT | AKI mild vs severe | chi-square | 0.3445 | 0.6171 |
| Ninja  score  (excluding  Iodixanol,  Polymyxin) | AKI none vs any | WRS | 0.3482 | 0.6182 |
| Vancomycin | Cancer dx | chi-square | 0.3511 | 0.6182 |
| Survived  60  days | AKI none vs any | chi-square | 0.3626 | 0.6330 |
| Contrasts | Cancer dx | chi-square | 0.3711 | 0.6424 |
| Ninja  score  (all  drugs) | Cancer dx | WRS | 0.3763 | 0.6460 |
| Cancer  diagnosis | AKI none vs any | chi-square | 0.3856 | 0.6545 |
| Age | Albumin <2.5 | WRS | 0.3876 | 0.6545 |
| BMT | Albumin <3.5 | chi-square | 0.3927 | 0.6577 |
| Survival  time  after  ICU  discharge | Albumin <2.5 | WRS | 0.4015 | 0.6670 |
| AKI  mild  vs  severe | Albumin <3 | chi-square | 0.4280 | 0.7004 |
| Max  phos | AKI none vs any | WRS | 0.4306 | 0.7004 |
| Race | AKI mild vs severe | chi-square | 0.4318 | 0.7004 |
| BMT | Albumin <2.5 | chi-square | 0.4457 | 0.7077 |
| Admission  Creatine | Cancer dx | WRS | 0.4464 | 0.7077 |
| Contrasts | AKI none vs any | chi-square | 0.4466 | 0.7077 |
| Vancomycin | AKI mild vs severe | chi-square | 0.4555 | 0.7163 |
| gender | Albumin <3 | chi-square | 0.4726 | 0.7375 |
| PICU  time  (days) | Albumin <3.5 | WRS | 0.4826 | 0.7475 |
| Nephrotoxicity Index | Albumin <2.5 | WRS | 0.4922 | 0.7567 |
| Max  CO2 | AKI mild vs severe | WRS | 0.5244 | 0.8002 |
| Min  phos | AKI none vs any | WRS | 0.5296 | 0.8022 |
| gender | Albumin <2.5 | chi-square | 0.5382 | 0.8071 |
| Min  K | AKI mild vs severe | WRS | 0.5407 | 0.8071 |
| BMT | AKI none vs any | chi-square | 0.5508 | 0.8163 |
| BMT | Albumin <3 | chi-square | 0.5594 | 0.8231 |
| Ethnicity | AKI mild vs severe | chi-square | 0.5698 | 0.8325 |
| Overall  survival  status | Albumin <3.5 | chi-square | 0.5843 | 0.8437 |
| Ethnicity (binary) | Cancer dx | chi-square | 0.5857 | 0.8437 |
| Mechanical  ventilation | Albumin <2.5 | chi-square | 0.6025 | 0.8496 |
| Min  K | AKI none vs any | WRS | 0.6115 | 0.8496 |
| Weight | Albumin <3 | WRS | 0.6128 | 0.8496 |
| Weight | Albumin <2.5 | WRS | 0.6152 | 0.8496 |
| Race | AKI none vs any | chi-square | 0.6206 | 0.8496 |
| Min  albumin | AKI mild vs severe | WRS | 0.6225 | 0.8496 |
| Survived  180  days | Albumin <2.5 | chi-square | 0.6237 | 0.8496 |
| Survived  1  year | Albumin <2.5 | chi-square | 0.6237 | 0.8496 |
| Age | Cancer dx | WRS | 0.6269 | 0.8496 |
| Mechanical  ventilation | Albumin <3.5 | chi-square | 0.6507 | 0.8761 |
| Total number of AKI drugs received | Albumin <2.5 | WRS | 0.6618 | 0.8853 |
| BMI | AKI none vs any | WRS | 0.6907 | 0.9114 |
| anitbiotics | AKI mild vs severe | chi-square | 0.6941 | 0.9114 |
| Contrasts | AKI mild vs severe | chi-square | 0.6946 | 0.9114 |
| AKI  mild  vs  severe | Albumin <3.5 | chi-square | 0.7050 | 0.9192 |
| Mechanical  ventilation | Cancer dx | chi-square | 0.7180 | 0.9302 |
| Min  protein | AKI mild vs severe | WRS | 0.7262 | 0.9350 |
| Weight | Albumin <3.5 | WRS | 0.7352 | 0.9403 |
| Survived  180  days | Albumin <3.5 | chi-square | 0.7440 | 0.9403 |
| Survived  1  year | Albumin <3.5 | chi-square | 0.7440 | 0.9403 |
| Overall  survival  status | AKI none vs any | chi-square | 0.7800 | 0.9659 |
| BMI  category | AKI none vs any | chi-square | 0.7815 | 0.9659 |
| Age | AKI none vs any | WRS | 0.7817 | 0.9659 |
| Mean  albumin | AKI mild vs severe | WRS | 0.7884 | 0.9659 |
| Age | Albumin <3 | WRS | 0.7902 | 0.9659 |
| AKI  None  vs  any | Albumin <3.5 | chi-square | 0.8017 | 0.9659 |
| Vancomycin | AKI none vs any | chi-square | 0.8039 | 0.9659 |
| Baseline  Creatine | AKI none vs any | WRS | 0.8043 | 0.9659 |
| BMI  centile | AKI mild vs severe | WRS | 0.8065 | 0.9659 |
| Mean  albumin | AKI none vs any | WRS | 0.8165 | 0.9721 |
| Max  CO2 | AKI none vs any | WRS | 0.8211 | 0.9721 |
| Min  protein | AKI none vs any | WRS | 0.8798 | 1.0000 |
| PICU  time  (days) | Albumin <2.5 | WRS | 0.8840 | 1.0000 |
| Min  albumin | AKI none vs any | WRS | 0.9248 | 1.0000 |
| Min  K | Cancer dx | WRS | 0.9274 | 1.0000 |
| Max  protein | AKI none vs any | WRS | 0.9372 | 1.0000 |
| Admission  Creatine | AKI none vs any | WRS | 0.9674 | 1.0000 |
| BMI  category | AKI mild vs severe | chi-square | 0.9685 | 1.0000 |
| Weight | AKI none vs any | WRS | 0.9960 | 1.0000 |
| Overall  survival  status | Albumin <2.5 | chi-square | 1.0000 | 1.0000 |
| ICU  survival | Albumin <2.5 | chi-square | 1.0000 | 1.0000 |
| Overall  survival  status | Albumin <3 | chi-square | 1.0000 | 1.0000 |
| ICU  survival | Albumin <3 | chi-square | 1.0000 | 1.0000 |
| ICU  survival | Albumin <3.5 | chi-square | 1.0000 | 1.0000 |
| Survived  30  days | Albumin <3.5 | chi-square | 1.0000 | 1.0000 |
| Survived  60  days | Albumin <3.5 | chi-square | 1.0000 | 1.0000 |
| gender | Albumin <3.5 | chi-square | 1.0000 | 1.0000 |
| Age | Albumin <3.5 | WRS | 1.0000 | 1.0000 |
| ICU  survival | AKI none vs any | chi-square | 1.0000 | 1.0000 |
| Survived  30  days | AKI none vs any | chi-square | 1.0000 | 1.0000 |
| Survived  180  days | AKI none vs any | chi-square | 1.0000 | 1.0000 |
| Survived  1  year | AKI none vs any | chi-square | 1.0000 | 1.0000 |
| Mechanical  ventilation | AKI none vs any | chi-square | 1.0000 | 1.0000 |
| anitbiotics | AKI none vs any | chi-square | 1.0000 | 1.0000 |
| Gender | AKI mild vs severe | chi-square | 1.0000 | 1.0000 |
| Ethnicity (binary) | AKI mild vs severe | chi-square | 1.0000 | 1.0000 |
| Overall  survival  status | AKI mild vs severe | chi-square | 1.0000 | 1.0000 |
| Survived  60  days | AKI mild vs severe | chi-square | 1.0000 | 1.0000 |
| Survived  180  days | AKI mild vs severe | chi-square | 1.0000 | 1.0000 |
| Survived  1  year | AKI mild vs severe | chi-square | 1.0000 | 1.0000 |
| Sepsis | AKI mild vs severe | chi-square | 1.0000 | 1.0000 |
| Immunosuppressants | AKI mild vs severe | chi-square | 1.0000 | 1.0000 |
| Overall  survival  status | Cancer dx | chi-square | 1.0000 | 1.0000 |
